# Synthetic strategies to access silacycles

**DOI:** 10.3389/fchem.2023.1200494

**Published:** 2023-06-15

**Authors:** Fengjuan Chen, Luo Liu, Wei Zeng

**Affiliations:** Key Laboratory of Functional Molecular Engineering of Guangdong Province, School of Chemistry and Chemical Engineering, South China University of Technology, Guangzhou, China

**Keywords:** cyclization, silacycles, transition-metal catalysis, photocatalysis, organocatalysis, silaarenes

## Abstract

In comparison with all-carbon parent compounds, the incorporation of Si-element into carboskeletons generally endows the corresponding sila-analogues with unique biological activity and physical-chemical properties. Silacycles have recently shown promising application potential in biological chemistry, pharmaceuticals industry, and material chemistry. Therefore, the development of efficient methodology to assemble versatile silacycles has aroused increasing concerns in the past decades. In this review, recent advances in the synthesis of silacycle-system are briefly summarized, including transition metal-catalytic and photocatalytic strategies by employing arylsilanes, alkylsilane, vinylsilane, hydrosilanes, and alkynylsilanes, *etc*. as starting materials. Moreover, a clear presentation and understanding of the mechanistic aspects and features of these developed reaction methodologies have been high-lighted.

## 1 Introduction

**FIGURE 1:**
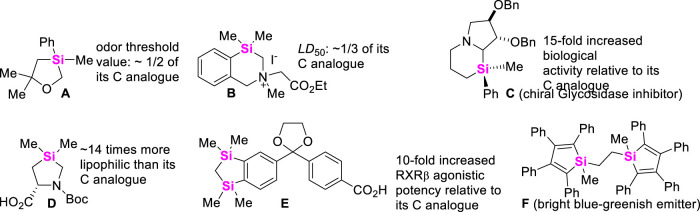
The difference in of biological activity and photophysical properties between the representative silacycles and their all-carbon analogues.

Nonmetallic Si-element which constitutes almost 30% of the mass of earth´s crust in silica and silicates exists extensively in our planet. As is well-known that aza-, oxa-, or thia-organic molecules are commonly encountered in many natural products and pharmaceuticals, but sila-organic molecules are not readily available in nature. Compared with all-carbon organic compounds, sila-analogues generally possess unique bioactive and photophysical properties (Förster et al., 2014; Pujals et al., 2006; Lippert et al., 2009) due to that silicon element has larger covalent radius (*r*_Si_ vs. *r*_C_: 111 p.m. vs 67 p.m.) and less electronegativity (*χ*_Si_ vs. *χ*_C_: 1.74 ev vs. 2.50 ev) different from carbon atom (Allred and Rochow, 1958). For instance, some representative examples of therapeutically potential silacycles are described in Figure 1. However, incorporating Si-element into all-carbon skeletons and heterocyclic systems has not been well-established. The main reason is that extra 3d orbitals of silicon element easily interact with heteroatoms and metal ions to form hypervalent five- or six-coordinated intermediates (Breit et al., 2013; Shen et al., 2017), leading to decomposition of organosilicanes (Akiyama and Imazeki, 1997; Fleming and Winter 1998). Meanwhile, transmetalation between sila-compounds and metal catalysts can often occur to make the silane-based chemical reactions show poor chemoselectivity (Chen et al., 2021; Pawley et al., 2022). Therefore, the development of versatile strategies to access various organosilanes has recently aroused increasing concerns. To date, the scope, mechanism, and applications for intermolecular silylations of alkanes, alkenes, arenes, diazo-compounds, and alkynes, etc., have been widely explored, and the corresponding studies involved transition metal-catalysis and photocatalysis have also been summarized by different research groups (Cheng and Hartwig, 2015; Li et al., 2015; Xu et al., 2015; Van Hoveln et al., 2019; Keipour et al., 2017; Chatgilialoglu et al., 2018; Huang W S et al., 2022). However, no comprehensive review about synthetical strategies of silacycles through C-C bond and C-heteroatom bond formation reactions has been published. This review will focus on the assembly of unique structural silacyclical skeletons through intermolecular and intramolecular coupling-cyclization of alkylsilanes, arylsilanes, alkynylsilanes, vinylsilanes, and hydrosilanes, etc., with different coupling-reagents.

## 2 Synthetic strategy of silacycles via C-Si σ bond functionalization

C-Si σ bond is commonly encountered in many organic sila-molecules. Therefore, the development of C-Si bond cleavage-based transformation will provide a novel platform to assemble silacycles with high step-economy. Generally, C-Si bonds include strained C-Si σ bonds and unstrained C-Si σ bonds. The cleavage of C-Si bonds catalyzed by transition metal is still in its infancy because of the unmet challenges on reactivity, selectivity, and substrate scope.

### 2.1 Coupling-cyclization by catalytic cleavage of the strained C-Si σ bond activation

**SCHEME 1:**
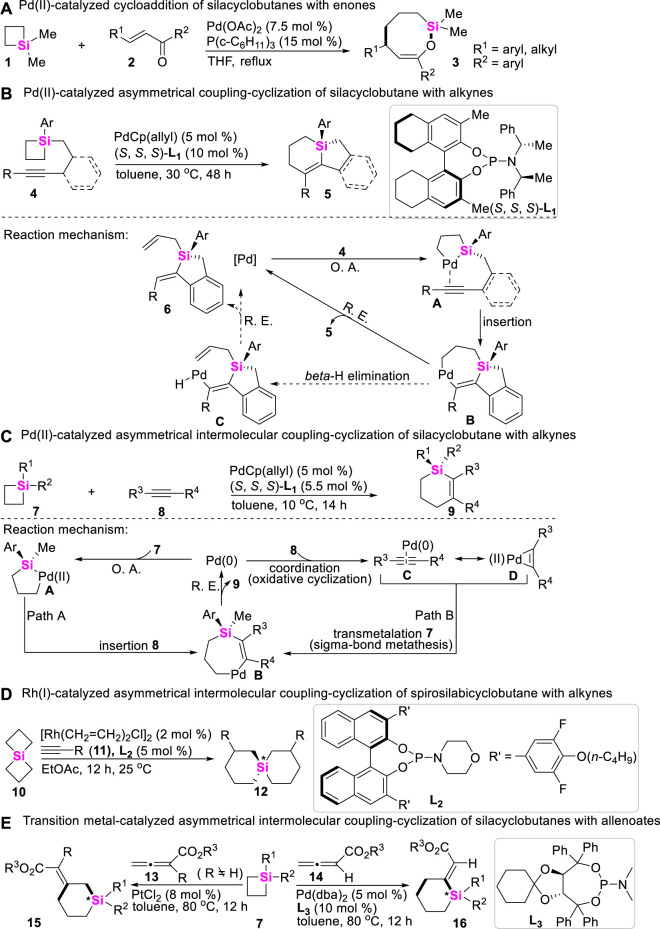
Transition metal-catalyzed coupling-cyclization of silacyclobutanes with alkenes, alkynes and allenoates.

The scope of strained C-Si bond cleavage is primarily restricted to the silacycles with a small ring size such as silacyclobutanes (SCBs), in which strain release can provide thermodynamic driving forces. In these regards, Oshima and Yorimitsu early reported a Pd-catalyzed intermolecular cycloaddition of SCBs with enones, in which coupling-cyclization of SCBs with enones under Pd-catalysis conditions could smoothly proceed via formal cycloaddition to yield the corresponding eight-membered ring (Hirano et al., 2008). The reaction was first triggered by the initial oxidative addition (O. A.) of SCBs to Pd (0) catalysts, producing palladasilacyclopentane which was then trapped by a, β-unsaturated ketones (Scheme 1A).

Possibly encouraged by Oshima’s work (Hirano et al., 2008), in 2011, Hayashi and Shintani developed a Pd-catalyzed enantioselective desymmetrization of SCBs through an intramolecular coupling-cyclization, in which alkyne-tethered SCBs **4** were used as starting material, and 5,5′,6,6′,7,7′,8,8′-octahydro-1,10-binaphthyl phosphoramidite (**L**_**1**_ **)** was utilized as a chiral ligand (Shintani et al., 2011). This transformation provided an efficient approach to access chiral silacycles **5** featuring tetraorganosilicon stereocenter (Scheme 1B). The overall process for this transformation starts with the oxidative addition of a C-Si bond of silacyclobutane from **4** to Pd (0) catalysts, giving 1-pallada-2-silacyclopentanes **A**. Intermediates **A** then undergo an intramolecular insertion of the alkyne to form 1-pallada-4-sila-2-cycloheptenes **B**, followed by the subsequent reductive elimination (R. E.) to produce compounds **5** along with regeneration of Pd (0)-catalysts. Meanwhile, β-H elimination from intermediate **B** gives alkenylpalladium hydride species **C**, and successive reductive elimination results in the formation of byproducts **6**.

Subsequently, Hayashi and Shintani further developed a Pd-catalyzed intermolecular desymmetrization of SCBs with alkynes almost using the same reaction conditions (Shintani et al., 2012), assembling Si-stereogenic 1-sila-2-cyclohexenes. The reaction mechanism possibly proceeds through Path A, in which oxidative addition of a C-Si σ bond of SCBs **7** to Pd (0) gives 1-pallada-2-silacyclopentane **A**. **A** species then undergo insertion of alkynes **8** to produce 1-pallada-4-sila-2-cycloheptenes **B**, the reductive elimination of **B** leads to the formation of products **9** along with regeneration of Pd (0)-catalysts. Alternatively, coordination of **8** to Pd (0) could precede cleavage of the C-Si σ bond of **7** as shown in Path B. Subsequent transmetalation (or σ-bond metathesis) of **7** can provide the same intermediates **B**, which eventually give silacycles **9** and Pd (0)-catalysts by reductive elimination (Scheme 1C). Of course, the detailed experiments and DFT calculation about this transformation were further performed by Xu, confirming that Path B (Scheme 1C) is reasonable (Zhang Q W et al., 2016). In 2021, the similar transformation of Rh/Cu-catalyzed coupling-cyclization of SCBs with arylpropiolate-type internal alkynes was also achieved by Xu (Wang X C et al., 2021). In 2022, Song developed a Rh (I)-catalyzed intermolecular asymmetric coupling-cyclization of siprosilacyclobutanes **10** with terminal alkynes **11** to produce spirosilabicyclohexenes **12** with up to 96% ee (Scheme 1D) (Chen et al., 2022). Meanwhile, Song still utilized allenoates **13** and **14** as coupling reagents to react with silacyclobutanes **7** to furnish 2- or 3-(*E*)-enoate-substituted silacyclohexenes **15** and **16** (up to 80% ee) in the presence of chiral phosphoramidite **L**_**3**_ (Scheme 1E) (Tang et al., 2022).

**SCHEME 2:**
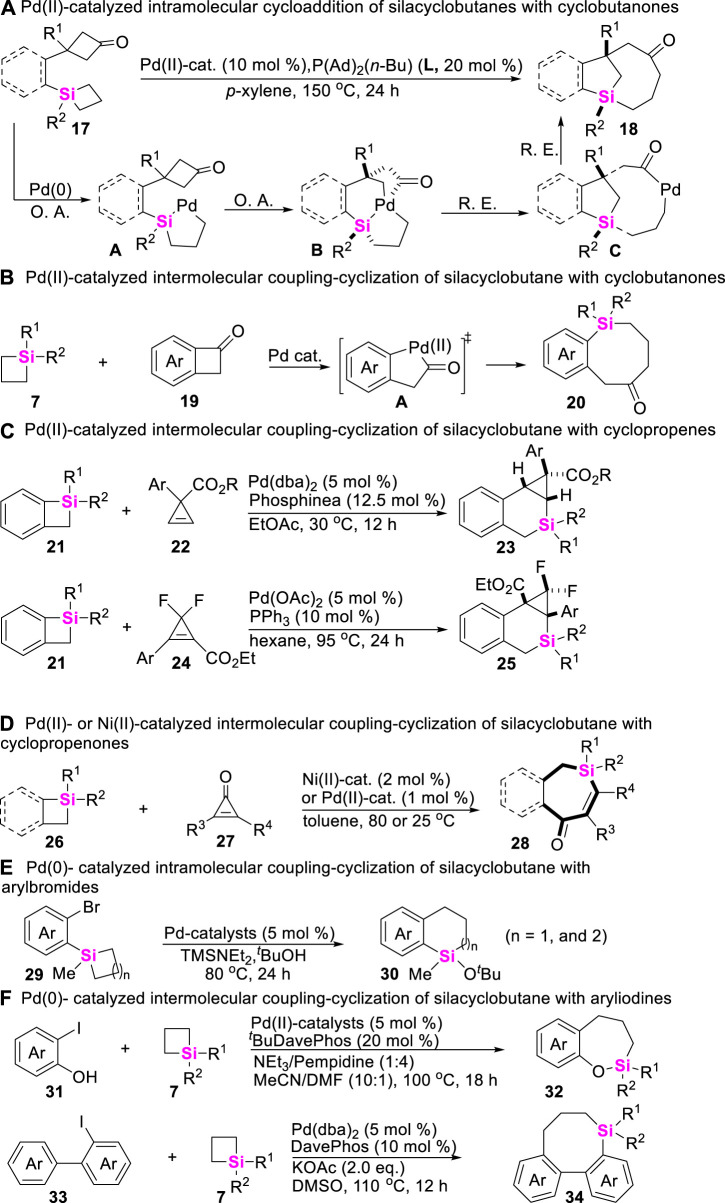
Transition metal-catalyzed coupling-cyclization of silacyclobutanes with cycloketones, cyclopropenes, and arylhalides.

Besides that a, β-unsaturated ketones and alkynes could be employed to couple with SCBs via C-Si σ bond cleavage, cycloketones, cyclopropenones, arenes, and trisylhydrazones were also demonstrated to be efficient coupling-partners. In this vein, Murakami successively developed Pd-catalyzed intramolecular and intermolecular coupling-strategies of cyclobutanones with SCBs to construct complex structural benzosilacycles through σ-bond exchange process. For the intramolecular coupling-cyclization of cyclobutanone-containing SCBs **17** (Ishida et al., 2014), the key step is involved the oxidative addition of Pd (0) onto Si-C σ bond of silacyclobutane moiety to generate silapalladacycles **A** (Scheme 2A). On the contrary, for the intermolecular coupling-cyclization of cyclobutanones **19** with SCBs **7** (Okumura et al., 2017), the control experiments confirmed that the oxidative addition of Pd (0) onto C-C bond of cycloketone moiety to generate palladacycle (II) intermediates (**A**) prefer to occur (Scheme 2B). Based on the similar reaction mechanism, Xu and co-workers developed a Pd (II)-catalyzed [4 + 2] annulation of cyclopropenes with benzosilacyclobutanes to rapidly assemble silabicyclo [4.1.0] heptanes **23** and **25** in which cyclopropenes include *gem*-difluorocyclopropenes **24** (Scheme 2C) (Wang W et al., 2021; Xu et al., 2022). Meanwhile, Zhao still found Pd (II)- or Ni (II)-catalytical system could rapidly enable the intermolecular coupling-cyclization of silacyclobutanes **26** with cyclopropenones **27** (Scheme 2D) and aryl halides **29**, **31**, and **33** (Schemes 2E–G), furnishing silacycles **28**, **30**, **32**, and **34** (Zhao et al., 2018a; Qin et al., 2020; Qin et al., 2021; Wang X et al., 2021).

**SCHEME 3:**
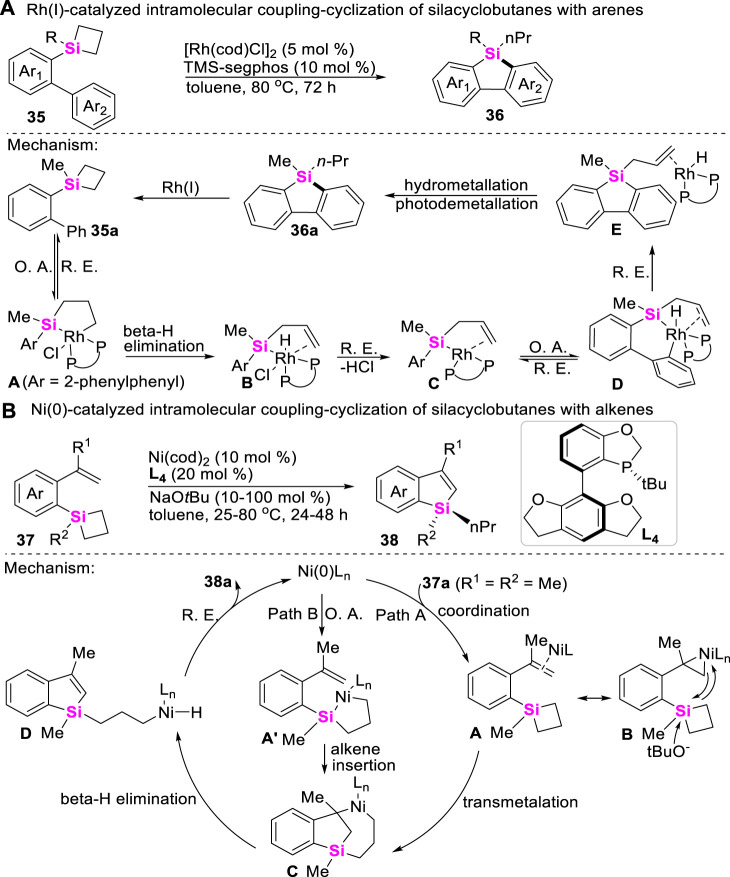
Transition metal-catalyzed coupling-cyclization of silacyclobutanes with Csp^2^-H bonds.

Meanwhile, the more challenging Rh-catalyzed intramolecular coupling-cyclization of SCBs with Csp^2^-H bonds has recently been achieved to make π-conjugated siloles with good regioselectivities by He (Zhang J et al., 2016). As shown in Scheme 3A, this transformation undergoes sequential C-Si bond and aryl Csp^2^-H bond activation process, and the catalytic cycle involves a rarely endocyclic β-hydride elimination of five-membered metallacycles **A**, which after reductive elimination gave rise to a Si-Rh^I^ species **B** that is capable of C-H activation. Apart from the Rh-catalysts, Zhao further developed a Ni (0)-catalyzed asymmetric intramolecular coupling-cyclization of alkene and SCBs to make enantioenriched silicon-stereogenic benzosiloles by utilizing *ortho*-vinylaryl silacyclobutanes **37** as substrates (Zhang et al., 2021). Two distinct pathways of this transformation are proposed. Path A begins with the coordination of the nickel (0) catalyst with alkene moiety to generate *η* ^*2*^–coordinated complex **A** or **B**, then followed by an alkoxide-promoted transmetalation, β-hydride elimination, and reductive elimination to produce the desired benzosilacycles **38**. In the contrast, Path B involves the oxidative addition of C-Si bond on SCB and sequential intramolecular insertion of an alkene moiety (Scheme 3B).

**SCHEME 4:**
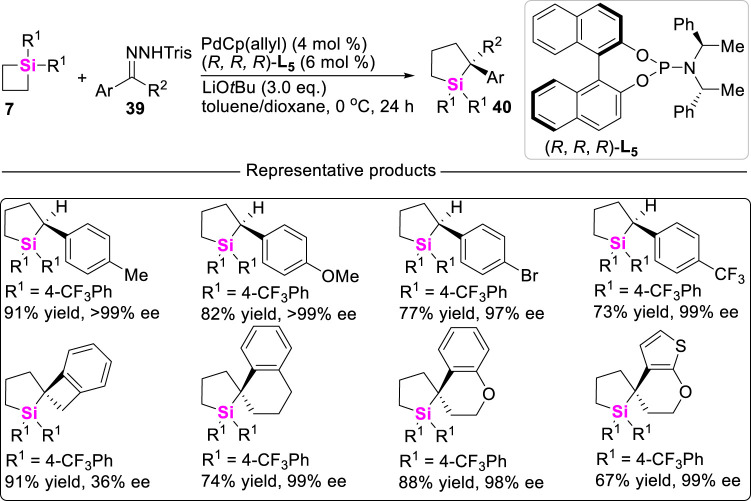
Pd (II)-catalyzed carbene insertion into C-Si bonds of silacyclobutanes.

More recently, Wang reported a highly effcient Pd (II)-catalyzed carbene insertion into C-Si bonds of SCBs **7** to deliver silacyclopentanes **40** with excellent enantioselectivity by using trisylhydrazones **39** as carbenoid precursors (Huo et al., 2021). This reaction features with wide substrate scope and high tolerance of functional groups. Mechanistic studies including DFT calculations suggest a catalytic cycle involving oxidative addition of Pd to strained C-Si bonds, carbenoid migratory insertion, and reductive elimination (Scheme 4). Moreover, the roles of the chiral ligand **L**_**5**_ in controlling the reaction enantioselectivity are also elucidated.

### 2.2 Coupling-cyclization by catalytic cleavage of the unstrained Si-C σ bond activation

**SCHEME 5:**
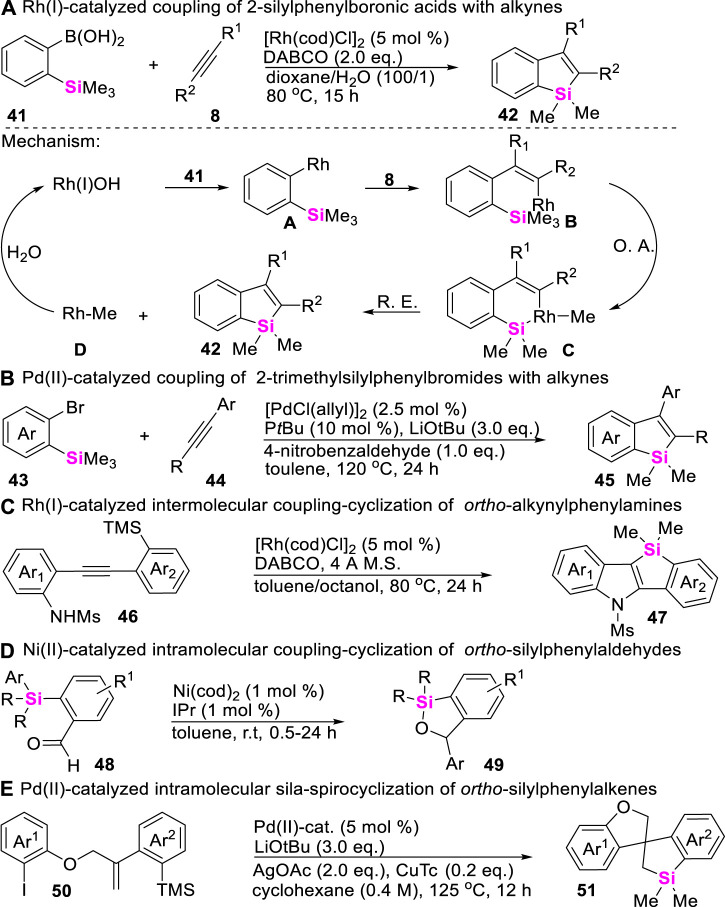
Transition metal-catalyzed unstrained C-Si bond activation.

In comparison with the strained C-Si bonds, unstrained C-Si σ bonds possess higher thermodynamic stability. Therefore, the inert C-Si bond cleavage generally requires the use of stoichiometric amounts of either organomagnesium (van Klink et al., 2002) or organolithium reagents (Yu et al., 2008) and harsh reaction conditions. Nevertheless, transition metal-catalyzed C-Si σ bond activation provides an alternative mild approach to enable C-Si σ bond cleavage. In these regards, Chatani first reported an Rh (I)-catalyzed benzosilole synthesis in 2009 through the coupling-cyclization of 2-silylphenylboronic acids with alkynes (Tobisu et al., 2009). The corresponding mechanism starts from the formation of arylrhodium intermediates **A**, generated by the transmetalation of arylboronic acids **41** to rhodium hydroxide Rh (I)OH, and adds across alkynes **8** to form the vinylrhodium intermediates **B**, which subsequently undergo oxidative addition at a trimethylsilyl group to afford intermediates **C**, producing benzosilole products **42** and methyl-rhodium **D** through reductive elimination. Meanwhile, protonolysis of **D** regenerates the catalytically active Rh (I)OH (Scheme 5A). By using the similar strategy, Xi, He, Ogoshi, and Zhao successively developed Pd-, Rh-, and Ni-catalyzed coupling-cyclization of unstrained C-Si σ bonds with alkynes (Schemes 5B, 5C), aldehydes (Scheme 5D), and alkenes (Scheme 5E), providing various synthetic methods to construct complex structural benzosiloles **45** (Liang et al., 2012) and **47** (Zhang et al., 2014), benzoxasiloles **49** (Hoshimoto et al., 2014), and spiro-silacycles **51** (Shi et al., 2022).

## 3 Synthetic strategy of silacycles via Si-H σ bond functionalization

Hydrosilanes belong to readily accessible organosilanes which possess versatile reactivity. So, the cross-coupling of hydrosilanes with alkanes, arenes, alkenes, and alkynes could provide an alternative strategy to form C-Si bonds. Among them, transition metal-catalyzed C-H/Si-H coupling and hydrosilylation of unsaturated hydrocarbons represent two main chemical transformations to assemble silacycles. Meanwhile, hydrosilanes including dihydrosilanes and monohydrosilanes are also the most common precursors of chiral tetraorganosilicons, and the development of synthetic methodology for chiral silacycles which are derived from hydrosilanes, has become more challenging.

### 3.1 Intramolecular coupling-cyclization of Si-H bonds with alkanes

**SCHEME 6:**
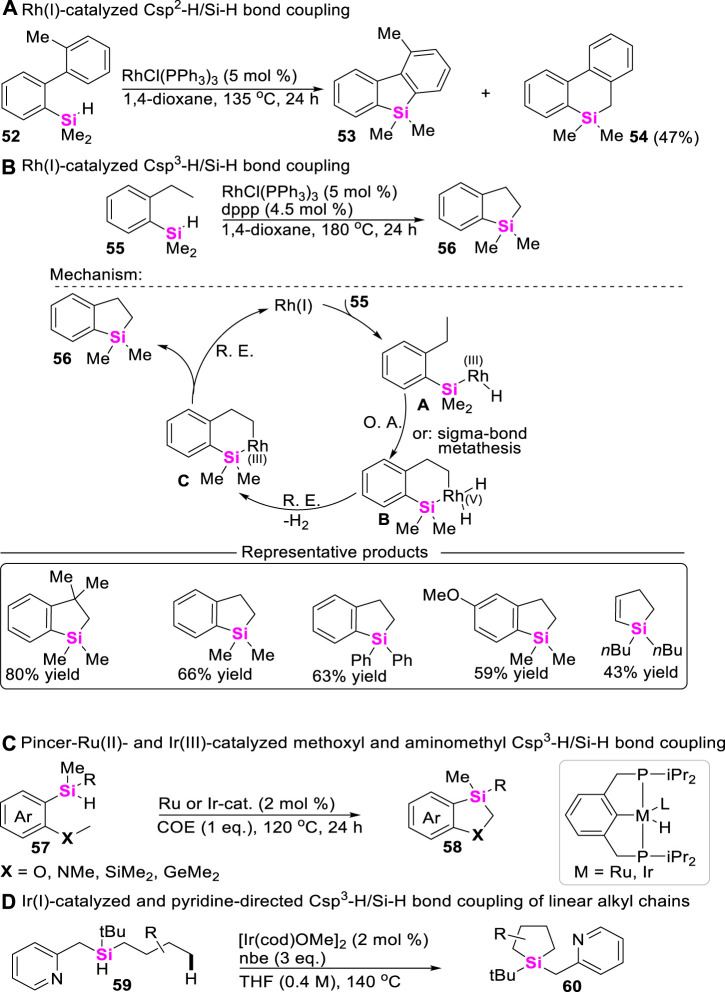
Transition metal-catalyzed Csp^3^-H/Si-H bond coupling reaction.

As is well-known, Csp^3^-H bond functionalization is one of the most useful and versatile strategies for constructing organic molecules. In comparison with Csp-H bond and Csp^2^-H bond, the reactivity of alkyl Csp^3^-H bond is inerter. Therefore, examples of Csp^3^-H/Si-H coupling reactions are very rare. The pioneer studies on transition metal-catalyzed Csp^3^-H/Si-H bond coupling reaction were mainly performed by Hartwig, but most of these transformations suffered from high reaction temperatures (135°C ∼ 200 C) (Tsukada and Hartwig, 2005). In 2010, Takai and co-workers reported Rh (I)-catalyzed Csp^2^-H/Si-H coupling reaction to produce the mixture of silafluorene **53** and dibenzo [b, d]saline **54** by using *ortho*-arylphenylsilanes including **33** as starting materials (Scheme 6A) (Ureshino et al., 2010). Subsequently, they further found that increasing the reaction temperature could chemoselectively enable Csp^3^-H/Si-H coupling under the same catalytical system, giving dibenzo [b, d]silane **56** as a sole intermolecular coupling-cyclization product by using *ortho*-alkylphenylsilanes as starting materials (Kuninobu et al., 2013a). The proposed mechanism was involved in the oxidative addition of the Si-H bond to Rh (I)-catalysts, then undergoing Si-H bond activation and σ-bond metathesis to form cyclorhodiumates **B** and **C**, respectively. Subsequently, reductive elimination of intermediate **B** or **C** produces benzosilacycle **56** and regenerates the Rh (I)-catalyst (Scheme 6B). To our satisfaction, Takai continued to optimize these reaction parameters by utilizing phosphorus ligand and 3,3-dimethyl-1-butene as additional additives, significantly lowering the reaction temperatures from 180°C to 50 C (Murai et al., 2015).

Possibly encouraged by Takai’s work, Huang further explored the synthetic approach to access 1,3-sila-heterocycles through methoxyl or aminomethyl Csp^3^-H/Si-H coupling of arylalkylhydrosilanes **57** (Fang et al., 2017), and found that PCP Pincer-Ru(II)- and PCP Pincer-Ir (III)-catalyzed intramolecular cyclization could rapidly assemble 1,3-sila-heterocycles **58** via intermolecular σ-bond metathesis process (Scheme 6C). As for the more challenging Csp^3^-H/Si-H coupling of trialkylhydrosilanes **59**, Gevorgyan developed an Ir(I)-catalyzed and pyridine-chelation-assisted Csp^3^-H silylation strategy of an unactivated C (sp^3^)–H bonds to produce silolanes **60** with good to excellent yields (Ghavtadze et al., 2014), in which different linear alkyl chains were well-tolerated in this reaction conditions (Scheme 6D).

**SCHEME 7:**
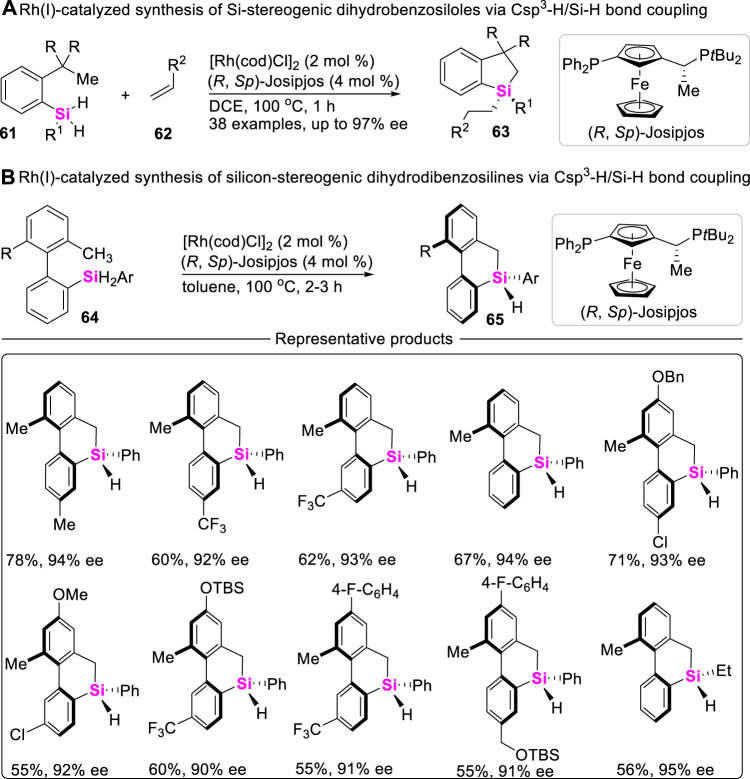
Transition metal-catalyzed asymmetric Csp^3^-H/Si-H bond coupling reactions.

Although chiral Si-atoms are not naturally occurring, these organosilanes show great application potential, especially in the field of life sciences and material chemistry. To date, the approach to access chiral sila-compounds through Csp^3^-H/Si-H coupling reaction is very limited. Albeit Takai ever achieved an asymmetric Csp^3^-H silylation toward silicon-stereogenic center, the corresponding enantiomeric excess (ee) values of spirosilabiindanes did not exceed 40% (Murai et al., 2015). Recently, He utilized Rh (I)/chiral Josiphos-catalytic system, successively realized intramolecular asymmetric Csp^3^-H/Si-H coupling-reaction of dihydrosilanes, constructing silicon-stereogenic dihydrobenzosiloles (Scheme 7A) (Yang et al., 2020) and dihydrodibenzosilines (Scheme 7B) (Guo et al., 2021) with excellent enantioselectivity (up to 97% ee values).

### 3.2 Intramolecular coupling-cyclization of Si-H bonds with arenes

**SCHEME 8:**
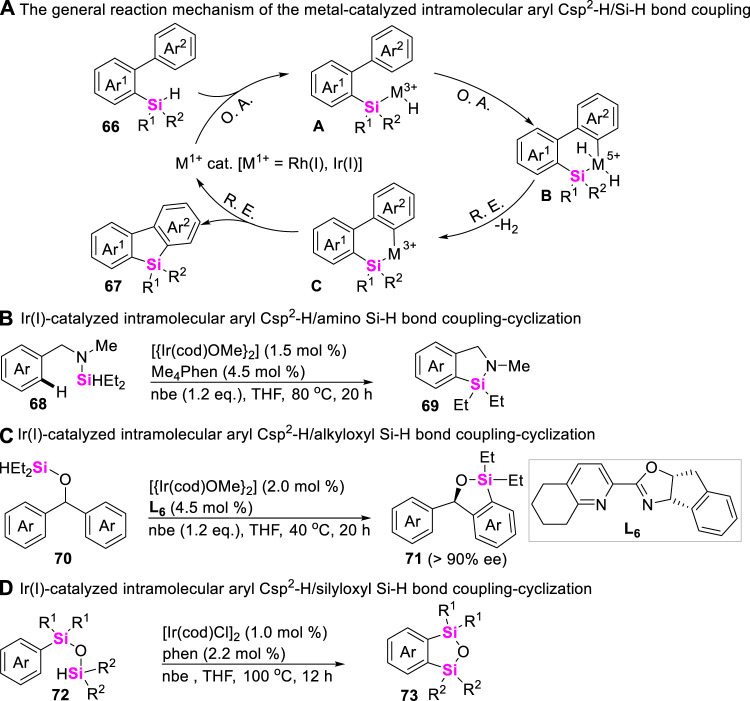
Transition metal-catalyzed intramolecular aryl Csp^2^-H/Si-H bond coupling reactions.

Silicon-containing π-conjugated molecules can be utilized in the areas of electro- and photo-luminescence, the intramolecular coupling-cyclization of aryl Csp^2^-H bonds with Si-H bonds has therefore been widely explored to furnish silaarenes. Usually, the present synthetic methods to access benzosilacycles are mainly focused on the set-up of different reaction systems, including arylsilanes and catalysts. The reaction mechanism was generally involved in the oxidative addition of low-valent metal ions including Rh (I) and Ir (I) to Si-H bond of hydrosilanes **66**, followed by the sequential aryl Csp^2^-H bond activation and reductive elimination process to afford diverse silaarenes **67** (Scheme 8A). The earlier study about transition metal-catalyzed aryl Csp^2^-H/Si-H coupling-reaction was reported by Takai in 2010 (Ureshino et al., 2010). Takai and co-workers utilized RhCl(PPh_3_)_3_ as catalysts, and employed biarylmonohydrosilanes as substrates to rapidly assemble silafluorenes through Rh (I)-catalyzed double activation of Si-H and C-H bonds with dehydrogenation. Since then, Hartwig (Li et al., 2014), Shi (Su et al., 2017), Zhao (Zhao et al., 2018b), and Xu (Lin et al., 2017) also realized Ir (I)-catalyzed intramolecular dehydrogenation-coupling of aryl Csp^2^-H bonds with monohydrosilanes **68**, **70**, and **72**, producing azo-silacycles **69** (Scheme 8B), oxa-silacycles **71** (Scheme 8C) and cyclic disiloxanes **73** (Scheme 8D), respectively.

**SCHEME 9:**
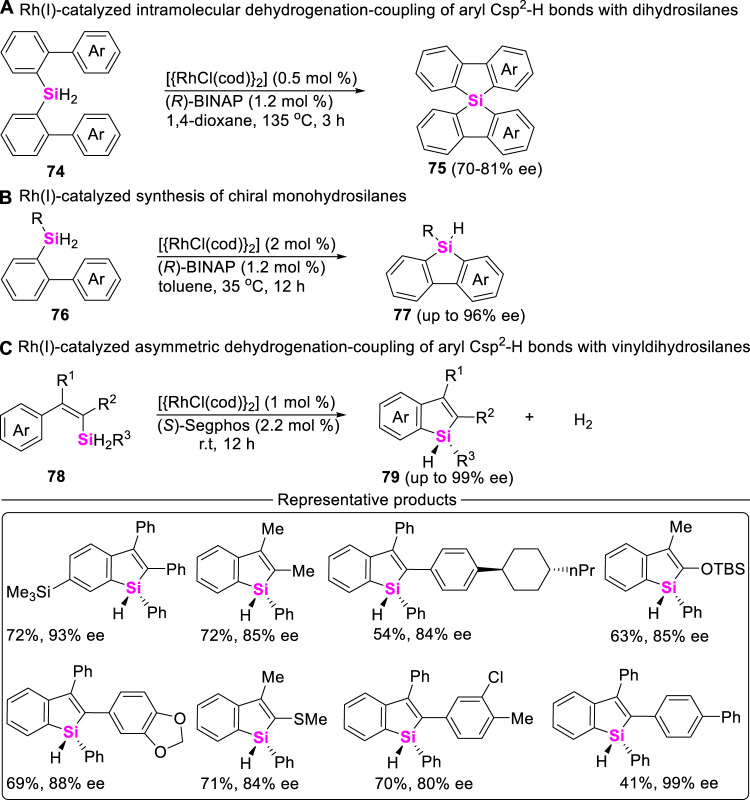
Rh-catalyzed intramolecular asymmetric dehydrogenation-coupling of aryl Csp^2^-H bonds with dihydrosilanes.

Of course, Takai still found Rh (I)-salts possess excellent catalytical activity to allow for dehydrogenation-coupling between aryl Csp^2^-H bonds and dihydrosilanes. For example, in 2013, Takai and coworkers developed an intramolecular asymmetric coupling-cyclization of bis (biphenyl)bihydrosilanes **74** in the presence of {[RhCl(cod)]_2_}] and chiral (*R*)-binap (Kuninobu et al., 2013b), providing spirosilabifluorenes **75** in 73%–95% yields (Scheme 9A). Encouraged by this work, W. He (Scheme 9B) and C. He (Scheme 9C) further realized the construction of stereogenic silicon benzosilacycles by using aryldihydrosilanes **76** and vinyldihydrosilanes **78** as starting material in the presence of Rh (I)-catalysts and chiral diphosphine ligands, excellent enantioselectivities of chiral benzosilacompounds **77** and **79** were up to >99% ee (Ma et al., 2021; Yuan et al., 2021).

**SCHEME 10:**
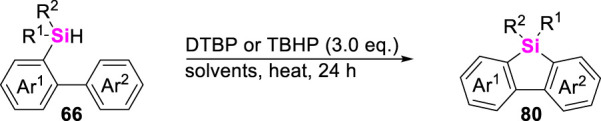
Oxidant-promoted intramolecular dehydrogenation-coupling of aryl Csp^2^-H bonds with hydrosilanes.

Besides that transition metal Rh (I)- and Ir (I)-catalysts could efficiently enhance the cross-coupling of aryl Csp^3^-H bonds with hydrosilanes, Studer and Li also found that oxidants-promoted aryl Csp^2^-H bond/Si-H coupling could easily occur to deliver benzosilacycles **80** in good reaction conversions (Scheme 10). This transformation is generally involved in the homolysis of oxidants such as DTBP and TBHP to produce alkoxyl radicals, which then abstracted the H atom from monohydrosilanes to form Si-centered radicals. Finally, the radical-cyclization between Si-centered radicals and arenes gave 9-silafluorene skeletons (Leifert and Studer, 2015; Xu et al., 2015).

### 3.3 Intramolecular coupling-cyclization of Si-H bonds with alkenes and alkynes

**SCHEME 11:**
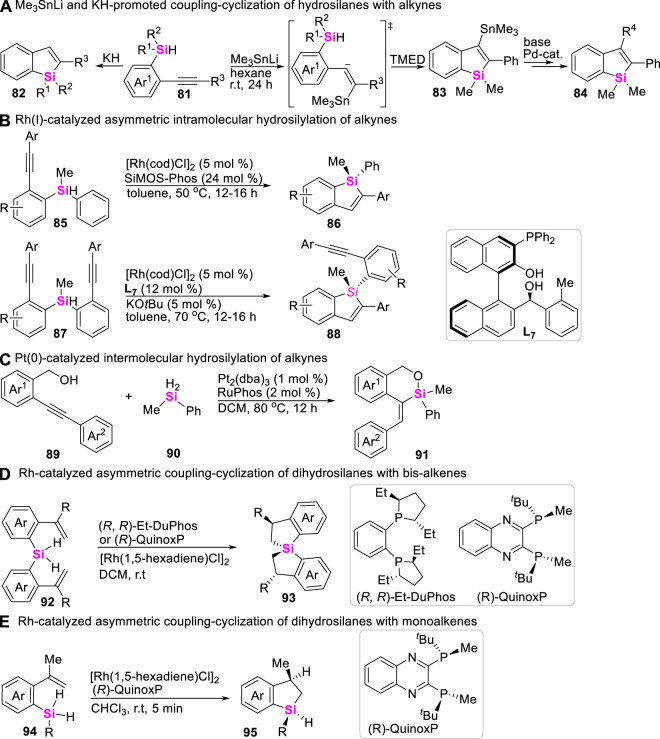
Intramolecular coupling-cyclization of Si-H bonds with alkenes and alkynes.

Despite the rich history of the synthesis of silacycles, the method of intramolecular coupling-cyclization of hydrosilanes with alkenes or alkynes has been significantly lacking. To date, only Nakamura in 2008 reported a Me_3_SnLi-promoted intramolecular coupling-cyclization of hydrosilanes with alkynes to produce benzosiloles (Ilies et al., 2008), in which nucleophilic addition of Et_3_SnLi to alkynes was involved (Scheme 11A). Subsequently, Nakamura further found that strong base KH could also promote the same transformation by using (2-alkynylphenyl)monohydrosilanes as substrates (Scheme 11A) (Ilies et al., 2009). By utilizing the similar substrates, Xu realized a Rh-catalyzed dynamic kinetic asymmetric intramolecular hydrosilylation with “silicon-centered” racemic hydrosilanes in the presence of chiral ligand SiMOS-Phos, providing silicon-stereogenic benzosiloles in excellent ee values (Scheme 11B) (Tang et al., 2020; Zeng et al., 2022). By the way, the Pt (0)-catalyzed intermolecular hydrosilylation of OH-containing acetylenes **89** with dihydrosilanes **90** was also achieved in 2018 by Xu to allow for assembling silyloxycycles and cyclic siloxanes **91** (Scheme 11C) (Long et al., 2018). In 2020, the transition metal-catalysis strategy has been gradually developed by Wang to assemble benzosilacycles through intramolecular coupling-cyclization of monohydrosilanes with alkenes. The intramolecular coupling-cyclization of bis-(alkenyl)dihydrosilanes (Chang et al., 2020) and mono-(alkene)dihydrosilanes (Huang Y H et al., 2022) could smoothly proceed in the presence of Rh (I)-catalysts and chiral ligands, producing spirosilabiindanes (Scheme 11D) and monohydro-benzosilacycles (Scheme 11E) with good to excellent ee values, respectively.

## 4 Synthetic strategy of silacycles via coupling-cyclization of vinylsilanes

**SCHEME 12:**
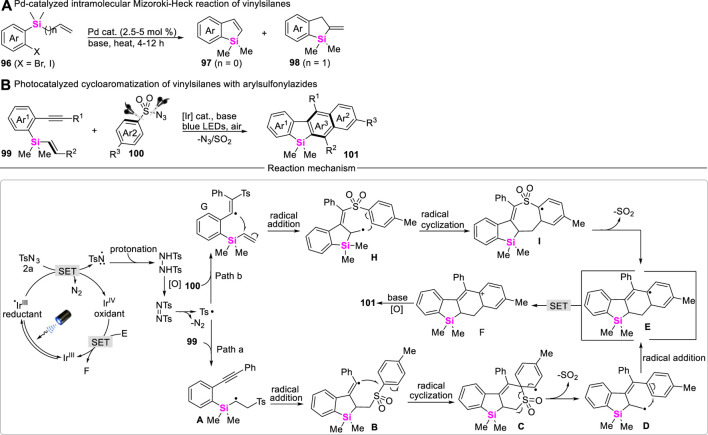
Coupling-cyclization of vinylsilanes.

Although vinylsilanes have been widely used in the Hiyama cross-coupling reaction to make C-C bonds with the release of the silyl group, transition metal-catalyzed Mizoroki-Heck reaction of vinylsilanes with aryl halides can form new C-C bonds at the β-position of vinylsilane and keep silyl moiety untouched. Therefore, the development of intramolecular Mizoroki-Heck coupling-cyclization of vinylsilanes may provide an efficient approach to access silacycles. Unfortunately, the examples involving vinylsilane which participated in the Mizoroki-Heck reaction are rarely reported. To date, only Xi and Teen successively reported Pd-catalyzed intramolecular coupling-cyclization of vinylsilanes with aryl Csp^2^-X bonds (X = Br and I) to assemble benzosilacycles (Scheme 12A) (Teng and Keese, 1999; Ouyang et al., 2012).

Aryl migration via Smiles rearrangement is a powerful tool for the synthesis of polycyclic arenes. However, the modes of radical Smiles rearrangement are very limited. More recently, Zeng reported a novel photo-catalyzed cycloaromatization of *ortho*-alkynylaryl vinylsilanes **99** with arylsulfonyl azides **100** for delivering naphthyl-fused benzosiloles **101**, various *ortho*-alkynylaryl vinylsilanes including *ortho*-alkynylaryl allylsilanes and arylsulfonyl azides are well-allowed for this reaction system (Chen et al., 2021). The corresponding reaction mechanism features a unique combination of cascade S-N/C-S bond cleavages and α-silyl radical Smiles rearrangement, in which silyl hyperconjugation effect (the so-called β-effect) plays a key role in controlling the regioselective coupling-cyclization (Scheme 12B). Post-synthetic applications indicate that these silaarenes show promising potential in luminescent materials.

## 5 Synthetic strategy of silacycles via coupling-cyclization of alkynylsilanes

**SCHEME 13:**
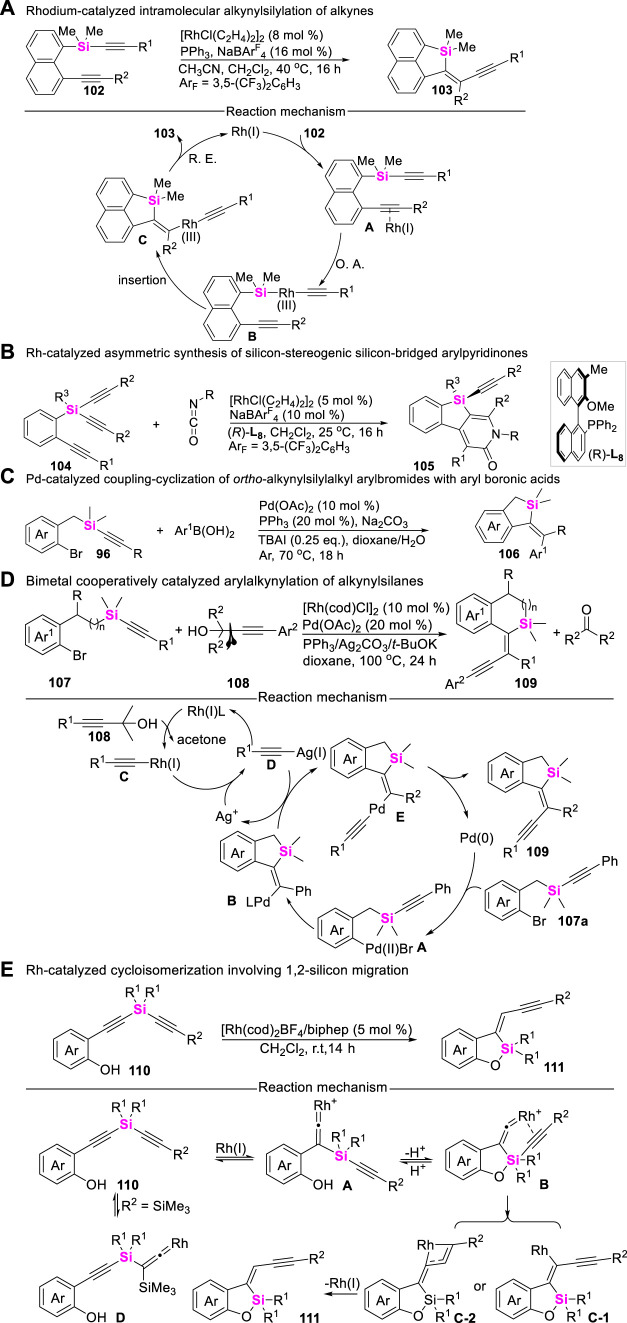
Coupling-cyclization of alkynylsilanes.

Alkynylsilanes belong to versatile synthons, possessing characteristic reactivity. Therefore, the development of alkynylsilane-based coupling-cyclization has aroused increasing concerns in the assembly of silacycles. In these regards, Nozaki utilized silicon-containing diynes **102** as substrates, explored the reactivity of Rh-catalyzed intramolecular alkynylsilylation of alkynes to produce enyne-functionalized dibenzosilacycles **103** (Shintani et al., 2015), this transformation was involved in sequential oxidative-addition, *syn*-insertion and reductive elimination process (Scheme 13A). Subsequently, Nozaki continued to design and synthesize prochiral triynes **104** and developed an Rh-catalyzed asymmetric [2 + 2 + 2] cycloaddition of silicon-containing prochiral triynes with isocyanates to afford silicon-stereogenic silicon-bridged arylpyridinones **105** (Shintani et al., 2016), high yields and enantioselectivities have been achieved by employing an axially chiral monophosphine ligand (Scheme 13B). Meanwhile, transition metal-catalyzed intramolecular coupling-cyclization of alkynylsilanes with aryl Csp^2^-X bonds (X = Br, I, etc.,) could also provide an alternated approach to access silacyclies. Possibly encouraged by the research work from Teen (Teng and Keese, 1999) and Ouyang (Ouyang et al., 2012) groups, Donnard utilized *ortho*-alkynylsilylalkyl aryl halides as substrates, realized the Pd (II)-catalyzed of coupling-cyclization of alkynylsilanes **96** with aryl boronic acids (Scheme 13C), rapidly assembling vinylation benzosiloles **106** (Wagner et al., 2017). More recently, Zeng developed an efficient Pd/Rh- cooperatively catalyzed arylalkynylation of *ortho*-alkynylsilyalkylaryl halides **107** with a-alkynylalcohols **108**, merging an alkynylidene moiety into benzosilacycle **109** (Chen et al., 2021). The corresponding mechanistic investigations demonstrated that the relay trimetallic transmetalation played a pivotal role in governing this transformation (Scheme 13D).

On the other hand, alkynylsilanes can also undergo 1,2-silicon migration under transition metal-catalytic systems (Kanno et al., 2016). Employing these reaction characteristics, Tanaka reported an Rh(I)/biphep complex catalyzeed cycloisomerization of 2-(alkynylsilyl-ethynyl)phenols **110**, leading to the formation of alkynylmethylidene-benzoxasiloles **111** through concomitant silicon and carbon migration (Namba et al., 2017). This novel cycloisomerization possibly proceeds via the formation of Rh-vinylidenes through 1,2-silicon migration, followed by 1,3-carbon (alkyne) migration via the formation of hypervalent silicon centers (Scheme 13E).

## 6 Synthetic strategies of silacycles via Si-Si σ bond activation of disilanes

**SCHEME 14:**
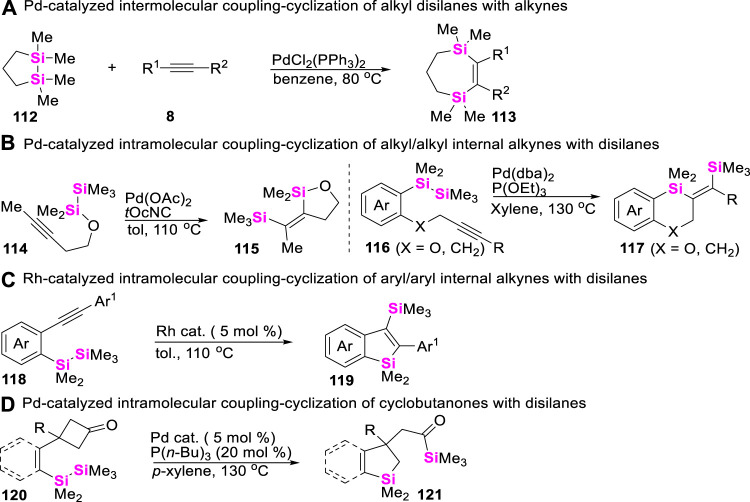
Coupling-cyclization of disilanes with alkynes and cyclobutanones.

The relatively weak Si-Si σ bond (ca. 54 kcal/mol) (Sanderson, 1983) suggests that disilanes could be converted into Si-M^n + 2^-Si species via low-valent metal-based oxidative addition. Thus, the addition of intermetallic Si-M^n + 2^-Si σ-bonds to alkynes can provide an efficient route for the preparation of silacycles. In these regards, the first example was evidenced by Sakurai et al., 1975, that a strained cyclic disilane **112** can react with activated alkynes **8** in the presence of Pd(II)-catalysts to provide disilyla-cycloheptenes **113** (Scheme 14A). Encouraged by this pioneering work, different disilanes were successively designed and synthesized to react with alkynyl moieties via intramolecular coupling-cyclization. For examples, Ito and Matsuda reported Pd-catalyzed intramolecular *syn* bis-silylation of alkyl/alkyl internal alkynes (Ito et al., 1991; Ahmad et al., 2017), providing straightforward access to a large set of *syn*-disilylated olefins **115** and **117** (Scheme 14B). In 2012, Matsuda and co-workers (Matsuda and Ichioka, 2012) utilized Rh (I)-catalysts to enable an intramolecular bis-silylation of aryl/aryl internal alkynes **118** into silylbenzosilanes **119** (Scheme 14C). Of course, apart from the coupling-cyclization of disilanes with alkynes, Pd-catalyzed intramolecular σ-bond metathesis between disilanes with cyclobutanones was also investigated by Murakami (Ishida et al., 2012) to furnish an acylsilane-tethered silaindane skeletons **121** (Scheme 14D).

## 7 Synthetic strategy of silacycles via cross-coupling of 1,3-dienes with dichrolosilanes and intramolecular ring-closing methathesis of diallylsilanes

**SCHEME 15:**
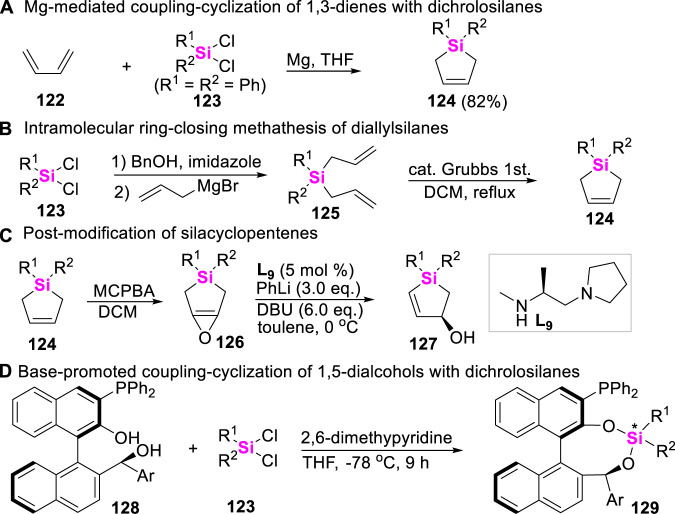
Synthetic strategies of silacyclopentenes.

Silacyclopentenes belong to versatile precursors of silacycles. In 2001, Kozmin reported an Mg-mediated cyclosilylation of butadiene **122** with dichrolodiphenylsilane **123** to produce silacyclopentene **124** (Scheme 15A) (Liu and Kozmin, 2001). Recently, Tomooka and Igawa developed an alternative approach to access silacyclopentenes **124** through successive diallylation of dichrolosilanes **123** and ring-closing methathesis of diallylsilanes **125** (Scheme 15B). These silacyclopentenes **124** could be further oxidized to furnish epoxide **126** by 3-chloro-peroxybenzoic acid (MCPBA), followed by post-modification to afford chiral silacyclopentenols **127** and other multi-functrionalized silacycles (Scheme 15C), featuring with interesting biological activity (Igawa et al., 2016; Igawa et al., 2017). By the way, it should be noted that dichrolosilanes could also easily react with 1,5-dialcohols **128** under base conditions to produce dioxasilanes **129** (Scheme 15D) (Bai et al., 2017).

## 8 Synthetic strategy of silacycles via cross-coupling of halomethylsilanes with unsaturated hydrocarbons

**SCHEME 16:**
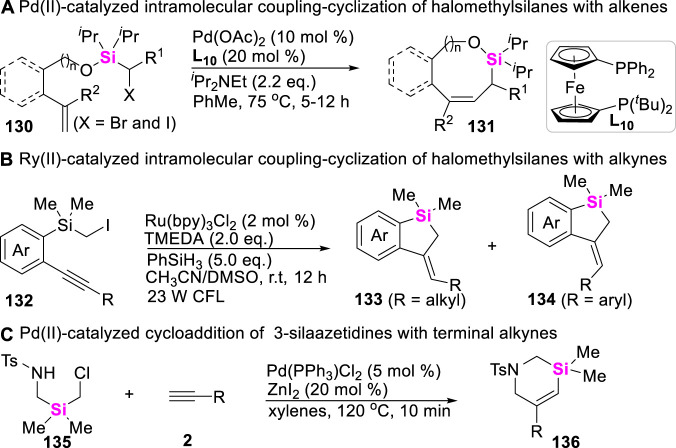
Coupling-cyclization of halomethylsilanes with unsaturated hydrocarbons.

To date, besides that these organosilicon compounds including silacyclobutanes, aryl/alkylsilanes, hydrosilanes, halosilanes, alkynylsilanes, vinylsilanes, allylsilanes and disilanes which have been successfully employed to couple with different coupling-reagents to make various silacycle skeletons, other novel silyl sources have also aroused wide concerns. In this regard, Gevorgyan designed and made halomethylsilyl ether-tethered alkenes **130**, and found that these halomethylsilanes can undergo intramolecular coupling-cyclization under Pd (II)-catalysis system to afford allylic silyloxycycles **131** via Heck reaction (Scheme 16A) (Parasram et al., 2014). Two years later, Song developed photo-catalyzed intramolecular coupling-cyclization of iodomethylsilanes with alkynes to produce 5-exo-cyclization products (Lin et al., 2016), in which alkyl-substituted internal alkynes lead to *Z*-benzosilolines **132**, aryl-substituted internal alkynes result in *E*-benzosiloline **134** (Scheme 16B). More recently, Song still further found that 3-silaazetidines can be easily prepared *in situ* from diverse air-stable precursors (RSO_2_NHCH_2_SiR_2_ CH_2_Cl **135**) (Scheme 16C), and this silyl source could easily undergo an intermolecular coupling-cyclization with terminal alkynes **2** in the presence of Pd (II)-catalysts, producing 3-silatetrahydropyridines **136** and diverse silaazacycle derivatives (Wang X et al., 2021).

## 9 Conclusion and perspectives

In summary, the methods for rapid assembly of silacycles are valuable to the fields of synthetic chemistry, material science, and biological chemistry. Therefore, the discovery of new reagents and new synthetic methodologies plays an important role in the development of organosilane chemistry. To date, the coupling-reaction between hydrosilanes with unsaturated hydrocarbons, alkanes, and arenes has been well-established; meanwhile, the studies on C-Si σ bond activation- and Si-Si σ bond activation-based coupling-cyclization have also obtained significant progress. By contrast, silylenoid-involved coupling-cyclization is very rarely reported. Although several silylenoid precursors such as di-*tert*-butyldiazidosilanes (Welsh et al., 1988), diamidodichlorosilanes (Denk et al., 1994), and cyclohexene-derived silacyclopropanes (Driver et al., 2002) have been reported, their applications in the construction of silacycles are very limited. Thus, a major goal for the future focus of this field is the development of silylenoid-based new organic reactions, which will be believed to provide a versatile strategy to access more complex structural silacycles.
